# Is hematoxylin-eosin staining in rectal mucosal and submucosal biopsies still useful for the diagnosis of Hirschsprung disease?

**DOI:** 10.1186/s13000-017-0673-9

**Published:** 2017-12-06

**Authors:** Suellen Serafini, Maria Mercês Santos, Ana Cristina Aoun Tannuri, Maria Claudia Nogueira Zerbini, Maria Cecília de Mendonça Coelho, Josiane de Oliveira Gonçalves, Uenis Tannuri

**Affiliations:** 1 Pediatric Surgery Division, Pediatric Liver Transplantation Unit and Laboratory of Research in Pediatric Surgery (LIM 30), University of Sao Paulo Medical School, Sao Paulo, Brazil; 2 Department of Pathology, University of Sao Paulo Medical School, Sao Paulo, Brazil; 3 Hospital das Clinicas HCFMUSP, Faculdade de Medicina, Universidade de Sao Paulo, Avenida Dr. Arnaldo 455, 4º andar sala 4108, CEP: 01246-903, São Paulo, SP Brazil

**Keywords:** Hirschsprung’s disease, Rectal biopsy, Acetylcholinesterase, Hematoxylin-eosin, Calretinin

## Abstract

**Background:**

Hematoxylin-eosin (HE) staining of a full-thickness rectal wall fragment is classically used for the diagnosis of Hirschsprung disease (HD). However, this technique requires large fragments for a better diagnosis. Additionally, the histochemical and immunohistochemical methods of staining small fragments of rectal mucosal and submucosal biopsies are not available in all centers. Therefore, the possibility of diagnosing HD through HE staining in these biopsies could be a valuable alternative for centers that do not have more specific techniques. The objectives of the current investigation were to evaluate the concordance of the results obtained by HE staining and the calretinin method with acetylcholinesterase (AChE) activity in fragments of mucosa and submucosa in the diagnosis of HD.

**Methods:**

For this study, 50 cases from our laboratory were selected. The tissue material was embedded in paraffin. Sixty levels of each fragment were utilized for HE, and the other 3 levels were used for calretinin. These slides were analyzed under the microscope, photographed and classified as either positive for HD when no ganglion cells were found with nerve trunks present or as negative when ganglion cells were found. The results from reading the slides were compared with those of AChE.

**Results:**

Of the 50 cases evaluated by the HE technique, only 5 contradicted the diagnosis based on AChE, with a Kappa value of 0.800 and an accuracy of 90%. In the comparison between calretinin and AChE, 8 cases were discordant, with a Kappa value of 0.676 and an accuracy of 84%.

**Conclusions:**

The concordance of results from AChE and HE methods was satisfactory, allowing for the potential use of the HE method for fragments of mucosa and submucosa as a valid alternative in the diagnosis of HD. The immunohistochemical technique of calretinin did not show good agreement with the AChE activity in our study.

## Background

Hirschsprung disease (HD) is a congenital disease that is characterized by the lack of enteric neuron formation during the embryonic period, with the absence of ganglion cells and hypertrophy of nerve trunks in the terminal segment of the large intestine [1–3].

Children with HD may exhibit symptoms of intestinal sub-occlusion and abdominal distension that generally begin as early as the first days of life. The proposed treatment is resection of the affected segment and pull through of the normal colon [1, 4].

The disease was first described in 1886 by Harald Hirschsprung, but Whitehouse and Kernohan did not define the etiopathogenesis of HD until 1948 [4, 5].

A biopsy of the rectal wall is the method of choice for diagnosis because of the pathological characteristics of HD. However, the best histological method for staining the rectal biopsy samples remains controversial [4–6].

The classical technique that is used for analysis of rectal tissue pathology is paraffinization followed by staining with hematoxylin-eosin (HE), which enables the visualization of enteric ganglion cells; however, this procedure typically requires large rectal fragments. Due to this requirement, the application of HE staining is limited, particularly in newborns [4, 5].

Newer and more specific histological methods have recently emerged for the diagnosis of HD. One of the most commonly used histological techniques is the acetylcholinesterase (AChE) assay. This method requires a small frozen rectal fragment that contains only the mucosal and submucosal slices. This fragment may be obtained less invasively compared to the tissue sampling needed for HE staining. This histochemical technique uses the fact that children with HD have increased AChE activity in their rectal mucosal and submucosal biopsy fragments [7–9]. This method is considered the gold standard for the diagnosis of HD in our practice, and it exhibits an accuracy greater than 90%.

A novel technique known as calretinin staining has been used for the diagnosis of HD due to the recent introduction of immunohistochemical markers. Calretinin is a calcium-binding protein that labels ganglion cells in the submucosal region of normal patients. Previous studies have indicated that the accuracy of this method is greater than 90%. This method may also be performed on a small fragment of rectal mucosa and submucosa, similar to the AChE assay. However, calretinin staining is more complex and expensive than HE staining, and it is only available in a few medical centers in Brazil [10, 11].

We recently hypothesized that HE staining of rectal mucosal and submucosal fragments may be used as an alternative method to diagnose HD, particularly in medical centers without access to advanced histological methods, such as the AChE and calretinin assays. We assessed the diagnostic accuracy of HE staining using small rectal biopsy fragments to detect HD. We also investigated the accuracy of the calretinin assay for the diagnosis of HD and compared both techniques to the AChE assay.

## Methods

This retrospective study examined 50 paraffinized specimens that were stored in our laboratory. These specimens were from biopsies obtained to confirm the diagnosis of HD in patients who were treated at our institution between 2010 and 2015.

Two fragments of rectal mucosa and submucosa were collected from each patient. One fragment was frozen for diagnosis using the AChE assay, and the other fragment was embedded in paraffin and stored for future studies.

### Sample preparation for the AChE assay

The fresh fragment for the AChE assay was frozen in liquid nitrogen and sectioned in a cryostat at −12 °C (IEC CTF Microtome Cryostat – Damon, USA). Eighteen sections of 10-μm thickness were obtained per fragment. These sections were incubated in a Karnovsky and Roots solution and processed according to previously published procedures [12, 13].

### Sample paraffinization

The samples were fixed in 10% buffered formalin solution for 24 h. Fixed tissue was dehydrated and diaphanized in an automatic tissue processor (Lupe PT05, Brazil).

### HE staining

Sixty 3-μm sections were obtained from each paraffin block using a microtome (Leica RM2255, Germany) and stained with HE. Samples were immersed in xylene and alcohol, stained with hematoxylin for 5 min, stained with eosin for 3 min and re-immersed in alcohol and xylene. Slides were mounted using a synthetic resin (Entellan; Merck, Germany). Slices were made from number 1 (most superficial) to 60 (deepest).

### Calretinin

Three additional sections from each paraffin block were obtained to analyze the immunohistochemistry of calretinin.

Antigen retrieval was performed using citrate buffer in a Pascal pressure cooker set to a temperature of 125 °C and a pressure of 18 psi (Celerus RIPTIDE – Celerus Diagnostics, CA, USA). Endogenous peroxidase was blocked, and the samples were incubated overnight in a solution containing rabbit anti-human calretinin monoclonal antibodies (clone SP13, Spring, USA) at a dilution of 1:400. Samples were incubated in N-Histofine Simple Stain (DBS, USA). Samples were incubated in the chromogenic substrate diaminobenzidine (DAB, from DBS) and counterstained with hematoxylin. Slides were mounted using a synthetic resin (Entellan; Merck).

### Analysis of tissue sections

Two investigators (M.M.S. and S.S.) analyzed the HE- and immunohistochemically stained sections using a Nikon optical microscope (Eclipse 50i; Nikon, Japan). Samples were blinded to avoid bias.

**Fig. 1:**
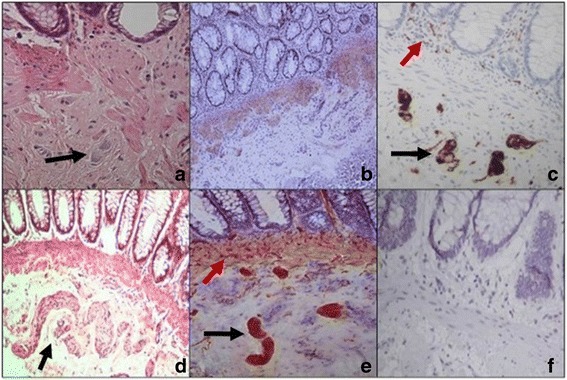
Histological patterns in HD disease - **a** HD negative by HE staining: ganglion cells located in the submucosal region (black arrow) at a magnification of 40×; **b** HD negative by AChE assay at a magnification of 10×; **c** HD negative by calretinin staining: ganglion cells (black arrow) and nerve fibers (red arrow) in the lamina propria at a magnification of 10×; **d** HD positive by HE staining: nerve trunks in the submucosal region (black arrow) at a magnification of 10×; **e** HD positive by AChE assay: nerve trunks (black arrow) and fibrils (red arrow) at a magnification of 20×; **f** HD positive by calretinin staining at a magnification of 10×

All 60 HE-stained sections were analyzed for the presence of ganglion cells. HD was confirmed if a ganglion cells were absent in all 60 sections. The image was saved if ganglion cells were observed, and the case was considered normal (Fig. 1 – a and d).

Staining of ganglion cells in the submucosa or thin fibrils in the lamina propria confirmed the absence of HD for the calretinin immunohistochemistry, and a lack of staining confirmed the HD diagnosis (Fig. 1 – c and f).

The final diagnosis for each case was reached via consensus of the two investigators.

### Statistical analysis

Diagnostic results using HE or calretinin staining were compared with the results from the gold standard AChE assay. These comparisons were made via the construction of contingency tables and analysis of the kappa index and chi-square statistical tests to evaluate sensitivity and specificity, respectively.

All analyses were performed using GraphPad InStat software (version 3.0; GraphPad InStat, Inc., San Diego, California, USA) with a significance level of 5%.

## Results

Twenty-six of the 50 cases were diagnosed as HD using the AChE assay as the gold standard for diagnosis. HD diagnosis was excluded in the other 24 cases.

### Comparison between AChE and HE

The results of the HE staining concurred with the AChE assay results in 90% of cases, with a kappa index of 0.800. Two of the five discordant cases were false positives, and three were false negatives (Table 1).

**Table 1:** Comparison of the diagnosis of HD using the HE vs. AChE (gold standard) assays

		HE		
		Not HD	HD	Total
AChE	Not HD	22	2	24
	HD	3	23	26
Total		25	25	50

**Fig. 2:**
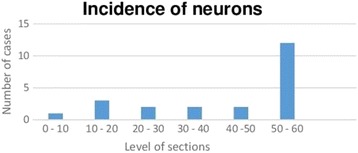
Location of ganglion cells using HE staining. Approximate slice

Tissue section examination identified the layers in which neuronal cells were present. The first histological section of the block corresponded to slice one, and sections were progressively obtained until slice 60 was reached, which was the deepest section of the block. In 54% of the 22 cases where ganglion cells were visualized they were observed only in the deepest sections (slices 50–60). In the remaining 46% of the cases, ganglion cells were distributed throughout the 60 slices (Fig. 2).

### Comparison between AChE and calretinin

The immunohistochemical examination of calretinin concurred with the results of the gold standard AChE assay in 84% of cases, with a kappa index of 0.676. Seven of the eight discordant cases were false positives, and one case was a false negative (Table 2).

**Table 2:** Comparison of the diagnosis of HD using calretinin immunohistochemistry vs. the AChE (gold standard) assay

		Calretinin		
		Not HD	HD	Total
AChE	Not HD	17	7	24
	HD	1	25	26
Total		18	32	50

The calretinin assay exhibited lower accuracy and specificity than the HE assay. The diagnostic accuracy was 84% vs. 90%, and the specificity was 70% vs. 92% for calretinin vs. HE, respectively (Table 3).

**Table 3:** Validation values of HE staining and calretinin immunohistochemistry diagnostic tests

	HE	Calretinin
Accuracy	96.0	84.0
Sensitivity	70.0	96.0
Specificity	78.0	70.0
Positive predictive value	94.0	78.0
Negative predictive value	84.0	94.0

## Discussion

HD is well-studied, but the histopathological aspects, pathogenesis and genetics of this disease have not been fully elucidated [3]. The current study reexamined the classic HE staining technique using smaller biopsy fragments, similar to the samples used in the AChE assay.

The efficacy of the HE staining method for the diagnosis of HD is controversial. Swenson [6] and Agrawal [9] used HE-stained rectal wall fragments to diagnose HD and observed a dispersed distribution of neurons in the submucosa. This distribution could hinder the diagnosis of HD, particularly when the histological analysis uses only a small number of sections from one fragment. Therefore, the authors disagree with the use of this method for the diagnosis of HD.

In contrast, Kapur et al. [1] demonstrated that the limitations of the method previously described by Swenson [6] and Agrawal [9] could be overcome by analyzing a larger number of sections from each fragment. However, there is no consensus in the current literature on the optimal number of sections to analyze for nerve plexus histopathology. Therefore, it is difficult to ascertain the most efficient examination methods. Some authors reported that only 15 sections are needed for evaluation, but others suggested up to 75 sections per fragment [1, 14].

Many centers currently use HE staining with the AChE or calretinin assay for the diagnosis of HD, and one exam complements the results of the other exam [14].

It seemed reasonable to analyze 60 sections per fragment in this study because of the large variation in recommendations. Our results demonstrated that 60 cuts were a good option because the ganglion cells were present in the deepest part of the paraffin block, between sections 50 and 60, in 54% of the cases. (Fig. 2).

The accuracy for HE staining was 90%, and the kappa value was 0.800 in the 50 cases that were studied. Only two of these 50 cases involved normal children who did not have ganglion cells present in the analyzed submucosal region. Three cases of HD exhibited some other structures that were confused with ganglion cells during the tissue section examination.

The accuracy obtained in this study was similar to other histological methods that are used in the diagnosis of HD, such as HE staining of total rectal wall biopsies and the AChE activity assay. Both of these techniques exhibit an accuracy over 90% [6, 8, 14].

Our tissue section examinations encouraged us to review the medical records of patients with discordant diagnoses between the HE and AChE assays to better understand the results. The colon segment that was surgically resected for the correction of the megacolon did not have ganglion cells present in the three patients who were diagnosed as normal using HE staining, which coincides with the diagnosis observed on the AChE assay.

The two patients who were diagnosed with HD using HE staining were not operated on because they received a normal diagnosis following the AChE assay. These children were clinically monitored and discharged after follow-up.

We consider the HE staining technique that includes at least 60 mucosal and submucosal sections to be a useful technique despite these discrepancies, but it should be tested in the clinical setting, as would be required for all diagnostic techniques.

Calretinin immunohistochemistry revealed a kappa value of 0.676 in our study, which is considered a good concordance index. However, the accuracy and specificity were only 84% and 70%, respectively. These values were lower than previous studies, in which accuracy and specificity were above 90% [10, 11, 15].

Our comparison between the calretinin and AChE assays revealed that eight of the 50 cases were discordant. Seven of these cases exhibited no immunohistochemical labeling, which led to a false positive diagnosis of HD. There was nonspecific labeling in one case, which resulted in a false negative diagnosis. Our results of the calretinin assay were worse than previous reports in the literature, but this method may be used routinely in the immunohistochemical diagnosis of HD.

## Conclusion

We conclude that HE staining of small fragments of the rectal mucosa and submucosa are a viable alternative method for HD diagnosis if at least 60 histological sections are analyzed.

## Abbreviations

AChE: Acetylcholinesterase
DAB: Diaminobenzidine
HD: Hirschsprung disease
HE: Hematoxylin-eosin
